# The influence of partnership centrality on organizational perceptions of support: a case study of the AHLN structure

**DOI:** 10.1186/1472-6963-6-141

**Published:** 2006-10-31

**Authors:** Spencer Moore, Cynthia Smith, Tammy Simpson, Sharlene Wolbeck Minke

**Affiliations:** 1 Centre for Health and Policy Studies, University of Calgary, Calgary, Alberta, Canada; 2 Alberta Healthy Living Network, Edmonton, Alberta, Canada; 3 Public Health Agency of Canada, Ottawa, Canada; 4 SWM Consulting, Edmonton, Alberta, Canada

## Abstract

**Background:**

Knowledge of the structure and character of inter-organizational relationships found among health promotion organizations is a prerequisite for the development of evidence-based network-level intervention activities. The *Alberta Healthy Living Network *(AHLN) mapped the inter-organizational structure of its members to examine the effects of the network environment on organizational-level perceptions. This exploratory analysis examines whether network structure, specifically partnership ties among AHLN members, influences organizational perceptions of support after controlling for organizational-level attributes.

**Methods:**

Organizational surveys were conducted with representatives from AHLN organizations as of February 2004 (n = 54). Organizational attribute and inter-organizational data on various network dimensions were collected. Organizations were classified into traditional and non-traditional categories. We examined the partnership network dimension. In- and out-degree centrality scores on partnership ties were calculated for each organization and tested against organizational perceptions of available financial support.

**Results:**

Non-traditional organizations are more likely to view financial support as more readily available for their HEALTR programs and activities than traditional organizations (1.57, 95% CI: .34, 2.79). After controlling for organizational characteristics, organizations that have been frequently identified by other organizations as valuable partners in the AHLN network were found significantly more likely to perceive a higher sense of funding availability (In-degree partnership value) (.03, 95% CI: .01, .05).

**Conclusion:**

Organizational perceptions of a supportive environment are framed not only by organizational characteristics but also by an organization's position in an inter-organizational network. Network contexts can influence the way that organizations perceive their environment and potentially the actions that organizations may take in light of such perceptions. By developing evidence-based understandings on the influence of network contexts, the AHLN can better target the particularities of its specific health promotion network.

## Background

Organizations are increasingly bringing their expertise and resources together to develop chronic disease prevention and health promotion programs in an integrated and collaborative manner[1]. In order to facilitate the development of integrated and collaborative approaches in Alberta, the *Alberta Healthy Living Network *(AHLN) was formed in July 2002. The AHLN's mission is to provide leadership for integrated, collaborative action to promote health and prevent chronic disease. Integrated approaches can be described as multi-sectoral, multi-strategic, multi-disease and multi-risk factor approaches to reduce chronic disease (*The Alberta Healthy Living Framework*. Edmonton: University of Alberta; 2004). With the increasing popularity of integrated, collaborative approaches, however, there exists the corresponding need to understand better the structure and effects of inter-organizational relations on organizational perceptions and actions.

Network analysis has become increasingly regarded in the health promotion literature as a reliable method for describing and assessing levels of community capacity and organizational collaboration [2-5]. The following paper is an exploratory network-analytic case study of the AHLN, focusing specifically on how the structure of inter-organizational partnership ties might influence organizational perceptions of funding availability. We hypothesize that organizations that are more central partners in the AHLN network will perceive a more supportive funding environment after controlling for organizational characteristics. In this regard, our analysis takes a structuralist approach to argue that inter-organizational networks influence organizational perceptions of the environment [6-8]. Organizational perceptions are important elements in organizational decision-making routines[7,9], and thus may influence organizational decisions and actions. If more central organizations perceive a more supportive environment, their actions, including their potential willingness to develop new or deepen current partnerships, may be influenced by this perception. By understanding the influence and effects of emergent inter-organizational linkages on organizational perceptions, health promotion professionals are better equipped to develop network-level intervention strategies that target the linkages that exist or should exist among organizations.

## Methods

### Sample

Telephone and face-to-face interviews were conducted with representatives from AHLN member organizations as of February 2004. Organizations self-identified a representative who was best qualified to discuss that organization's AHLN-related activities. In general, respondents worked in the area of healthy living, specifically around healthy eating, active living, or tobacco reduction. Their organizational roles included executive directors, managers, and service providers. Different organizational roles can potentially provide different descriptions of an organization's relationships [10]. Three organizations chose to respond in a group format. In these instances, two to four respondents collaborated to provide a single response on behalf of their organization. These organizations were all traditional health organizations, specifically regional health authorities. While group respondents may have potentially identified more partnership ties, we allowed the potential variation to occur so as to maintain a participatory approach in our network mapping endeavor. When we excluded these three organizations in secondary analyses, we observed no significant change in our findings. The survey response rate was 100% (n = 54), although not all questionnaire items were answered.

Formal network analytic methods were used to identify and measure the character and intensity of ties among organizations. For the network modules, organizational representatives referred to the list of AHLN members as they answered questions regarding their relationships with those organizations.

### Measures

#### Dependent Variable

To assess organizational perceptions of the funding environment, organizational representatives were asked to respond based on a four-point Likert scale from strongly disagree to strongly agree to the following statement: "Financial support for your organization's programs and activities in Healthy Eating, Active Living and Tobacco Reduction (HEALTR) is readily available." The question was designed to tap into an organization's perception of whether the funding environment was one in which they felt that they could readily obtain the resources necessary to carry out their programs and activities.

#### Organizational Attributes

To maintain stable statistical estimates with the small sample size, the number of organizational-attribute variables was kept to a minimum in this analysis. Two organizational characteristics were included in the analysis: i) organizational type and ii) size. Organizational size was based on the total number of employees hired by an organization in either a full- or part-time capacity.

For organizational type, each organization was classified as belonging to either the traditional or non-traditional health sector (Minke S.W. and Simpson T. *AHLN Network Mapping: Report on Intersectoral Involvement in the Alberta Healthy Living Network*. Unpublished Report, September 2004). The criteria used to classify organizations were established in consultation with key stakeholders in the AHLN, and based on rules around membership, mandates, and action strategies for AHLN organizations (Minke S.W. and Simpson T. *AHLN Network Mapping: Report on Intersectoral Involvement in the Alberta Healthy Living Network*. Unpublished Report, September 2004). For example, the primary mandate of traditional health-sector organizations was to improve health status. Provincial and federal government health departments, regional health authorities, chronic disease prevention charities, and health professional associations were classified as traditional members of the AHLN (n = 31). These organizations varied in their health promotion activities, with some focusing on primary prevention (e.g., school health coalition), others concentrating on secondary prevention (e.g., chronic disease non-profit), and a few targeting tertiary prevention (e.g., renal program). In contrast, the mandate of non-traditional health-sector organizations did not explicitly include improving health status, although the value of health activities may have been incorporated into their agendas. The organizations deemed to be non-traditional were active living organizations (e.g., fitness centres), education departments (e.g., university, provincial government), recreation and sport organizations, aboriginal organizations, and private businesses (n = 23).

#### Network Variables

The value of inter-organizational partnership ties within the AHLN network was ascertained by asking organizational representatives the following question for each of the other AHLN members: "Do you have a partnership arrangement with (name of other AHLN member), if so, on a scale of 1–5 where 5 is critically valuable and 1 is marginally valuable, how would you rate your partnership with (name of other AHLN member) to the success of your work in Healthy Eating, Active Living, and Tobacco Reduction (HEALTR)?" The importance of such partnership ties for the overall work of the AHLN was determined through consultation with the Partnership Development and Community Linkages Working Group (PDCLWG) of the AHLN Coordinating Committee.

To measure organizational centrality, we used a Freeman degree measure. In this case, Freeman degree measures the organizations with the most ties to other organizations in the network and has been used in previous studies as an "index of potential communication activity"[11]. In directed networks, we may distinguish between sending, i.e., the partnerships that organization *x *reports having with others, and receiving ties, i.e., the partnerships that other organizations report having with organization *x*. Out-degree scores capture an organization's influence; while in-degree scores measure an organizations'popularity and prestige in a network[12]. The distinction is important since a contrast can exist between how organization *x *values its relationships with other AHLN members and how other organizations value their relationships with organization *x*. In- and out-degree scores were calculated using UCINET [13].

In addition to measuring organizational positions in the network, we also examined the percentage of tie homophily among traditional and non-traditional organizations. Tie homophily refers in this instance to the idea that traditional organizations may maintain a higher percentage of their network ties with other traditional organizations, and non-traditional organizations may maintain a higher percentage with other non-traditional organizations. Higher percentage tie homophily thus indicates less cross-organizational type diversity in an organization's network ties.

### Analysis

Using the statistical package SPSS, the analysis proceeded in three steps. First, we analyzed the distribution of organizational and inter-organizational variables for all AHLN members and then according to traditional or non-traditional organizational type. We examined if significant mean differences in our study variables existed between traditional and non-traditional organizations. Second, we used Pearson and Spearman-rho correlation analyses to examine significant associations among variables. Since there were no significant differences to report between Pearson and Spearman-rho correlation values, Pearson correlation analysis results are reported. Third, we constructed two ordinal logistic regression models. Model 1 regressed organizational-level perceptions of available financial support on two organizational characteristics. In model 2, we added our network measures of in-degree (prestige), out-degree(influence), and tie homophily to model 1. In secondary analyses, we also constructed a network effects model in which we included the term *ρWY *to adjust for autocorrelation between connected actors[14]. The weighting matrix *W *was based on i) organizations having equal levels of perceived support and ii) having direct reciprocal partnership ties. The basic premise is that the influence of other organizations' perceptions is strongest when those organizations perceive the same level of support and have reciprocal relationships. The network effects model allows adjustment for the influence of other organizations' perceptions on the ego organization's perception[15]. Secondary analyses found that the network effects term was not significant and did not alter the significance level of any variables in model 2. The regression procedure examines the odds of a variable predicting a higher organizational perception of support, i.e., agreeing or strongly agreeing with the statement that "financial support for your organization's programs and activities in Healthy Eating, Active Living and Tobacco Reduction (**HEALTR**) is readily available." Results are reported using maximum likelihood estimates.

## Results

**Figure 1:**
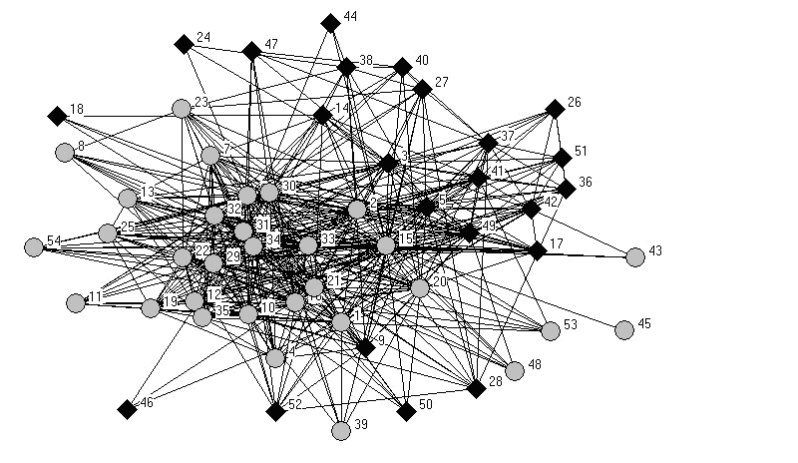
**Partnership Value Network**. Traditional and Non-Traditional Organizations: Traditional Organizations are represented by grey circles; Non-Traditional Organizations are represented by black diamonds.

Table 1 presents variable distributions for the AHLN population and specific organizational types. Significant average differences were found between traditional and non-traditional organizations in organizational size, budgetary priority, and partnership-value in- and out-degree scores, and tie homophily. Table 2 presents the Pearson correlation results. For brevity, we focus on the correlations between i) organizational type and the other study variables and ii) the network measures. Non-traditional health organizational status is negatively associated with in- (-.360, p < .01) and out-degree scores (-.384, p < .01) and percentage tie homophily (-.295, p < .05). Non-traditional organizations tend to be less influential and prestigious, and to maintain a lower percentage of their ties to other non-traditional organizations, compared to traditional organizations. Partnership in-degree is positively associated with partnership out-degree (.667, p < .01), organizational size (.360, p < .05), and perceptions of more readily-available support (.224, p < .05). In other words, more prestigious organizations tend also to be more influential, larger, and sense a higher degree of support. Using diagnostic procedures, we confirmed that the moderately high collinearity between in- and out-degree scores did not result in unstable coefficient estimation. Percentage tie homophily is only correlated with organizational status. Figure 1 shows the location of traditional and non-traditional organizations in the AHLN partnership network.

Table 3 provides the ordinal logistic regression results. In model 1, the positive and significant coefficient for non-traditional health organizations indicates that being a non-traditional health organization increases the probability that such organizations view funds as more readily available for their HEALTR programs and activities than traditional organizations (1.57, 95% CI: .34, 2.79). Organizational size had no apparent influence on organizational perceptions of funding support. Model 2 adjusts for the network characteristics of the AHLN. The positive and significant coefficient for partnership-value in-degree indicates that higher in-degree scores increase the likelihood that organizations will agree with the statement that financial support is readily available for their *HEALTR *programs and activities (.03, 95% CI: .01, .05). In other words, organizations that receive a greater number of partnership ties or receive more highly valued ties, i.e., identified as being important partners by others, are significantly more likely to perceive higher levels of financial support available. The influence of non-traditional organizational status on perceptions of support remain after adjusting for AHLN network features (1.55, 95% CI: .22, 2.89). Percentage tie homophily or its opposite tie heterophily across organizational types appears to have no direct influence on perceptions of support, although secondary analyses suggest that tie homphily may attenuate the influence of non-traditional organizational status. In secondary analyses, the network effects term was not significant nor did it alter the significance of the other variables in model 2. For this reason, the network effects model is not reported, although available upon request to corresponding author.

## Discussion

Our analysis of the AHLN suggests that both network and organizational characteristics influence member's perceptions of available support. There are three questions that our study's findings raise that require further elaboration: 1) why does in-degree and not out-degree partnership ties have an influence on organizational perceptions of support?; 2) why do non-traditional have higher perceptions of support than traditional organizations?; and 3) how might in-degree ties help traditional organizations create a more secure funding environment? First, we found that in-degree has a significant, positive influence on organizational perceptions of support. In other words, if an organization receives more ties, they report a higher perception of readily available financial support. These findings held when we also adjusted for social influence, or network effects, on organizational perceptions. Although organizations with higher in-degree scores tend also to have higher out-degree scores, an organization's sending ties do not have a significant association with organizational perceptions of support. Why would receiving partnership ties have an influence on perceptions of support while sending partnership ties do not? The simplest interpretation may be that partnership ties provide supportive resources that contribute to an organizations' general pool of available resources. Organizations receiving more support through partnership linkages would tend to perceive a more supportive environment. This is not the case with sending ties since they represent partnership relations in which resources are potentially flowing outwards from an organization. Organizational influence might emerge through partnership ties in which resources are sent but this does not appear to contribute to an organization's sense of support available through partnership ties.

Second, we found that non-traditional organizations, such as active-living centres, private business, and educational centres, were significantly more likely than traditional health organizations to view funding support as readily-available for their *HEALTR *programs. Although tie homophily did not have a direct influence on perceptions of support, non-traditional organizations do have a greater diversity in their ties across traditional and non-traditional organizational types. While our data do not allow us to confirm this empirically, non-traditional organizations may maintain more diverse networks across a range of other organizational types and domains, thus having more avenues of support for their activities than traditional organizations.

Third, we found that traditional health organizations have on average significantly lower perceptions of support, despite receiving on average more partnership ties in the AHLN network. In the case of traditional organizations, receiving partnership ties appears to increase their access to network resources, informational or financial. Since traditional health organizations are more explicitly tied to health-related mandates, i.e., their mandates specifically include "improving health status," such organizations may have a reduced range of overall activities and less access to diverse funding sources than non-traditional health organizations. In this sense, the greater development of partnership ties among traditional health organizations may represent an important organizational strategy that has helped such organizations buffer the potential funding insecurity surrounding their more-specialized organizational activities. Further research, including the use of longitudinal data, is required to confirm the potential factors that might help explain our present findings.

## Conclusion

While organizational characteristics are important, our study has shown how network environments also play a role in shaping the way organizations see the availability of support for their programs and activities. In studying the association among network structure, organizational characteristics, and perceptions, our analysis highlights the importance of receiving partnership ties in influencing organizational perceptions of readily available support. For traditional organizations, these receiving ties appear to be a particularly important mechanism in which such organizations develop or enrich their avenues of possible support.

Given the AHLN mission to provide leadership for integrated, collaborative action to promote health and prevent chronic disease, we see this exploratory study as encouraging the continued development of evidence-based health promotion activities and contributing to the use of network mapping activities to assess the dynamics of inter-organizational collaboration[16]. Although organizational perceptions are subjective, they are important elements in organizational decision-making routines[7,9]. By understanding better how these perceptions may be influenced by the structure of partnership ties that organizations find themselves embedded, we are better positioned to design network-level intervention activities and evaluate the impact of such interventions on organizational perceptions and the inter-organizational structure itself. Through future assessments of the AHLN member characteristics and network structure, we will be better able to examine how those characteristics and structures may change as organizations pursue their HEALTR programs and activities.

## List of Abbreviations

Alberta Healthy Living Network (AHLN)

Healthy Eating, Active Living and Tobacco Reduction (HEALTR)

Partnership Development and Community Linkages Working Group (PDCLWG)

## Competing interests

The author(s) declare that they have no competing interests.

## Authors' contributions

SM led the study design, analyses, and writing. CS, TS, and SWM assisted with the study and analyses. All authors helped to conceptualize ideas, interpret findings, and review drafts of the article.

## Pre-publication history

The pre-publication history for this paper can be accessed here:



## Figures and Tables

**Table 1:** Descriptive Statistics (by Organizational Type)

	**Variable**	**All Organizations**	**Traditional Health (n = 32)**	**Non-Traditional Health (n = 22)**
		Mean	Mean	Mean
**Organizational Characteristics**	Size	496.74 n = 50†	713.48** n = 29	197.43** n = 21
**Network Dimensions**	***Partnership Value***			
	In-Degree	53.12	65.25**	35.55**
	Out-Degree	53.12	66.78**	33.32**
**Network Ties**	Percentage Tie Homophily		.78*	.62*
**Organizational Perceptions**	Support	2.43, n = 51	2.26, n = 31	2.7, n = 20

1. p < .05, ** p < .01

† Lower sample sizes than 54 indicate missing responses on certain variables.

**Table 2:** Pearson Correlation Values

	Traditional Health	Size	PV In-Degree	PV Out-Degree	Percentage Tie Homophily
Traditional Health					
Size	.238 n = 50				
PV In-Degree	.360** n = 54	.360* n = 50			
PV Out-Degree	.384** n = 54	.226 n = 50	.667** n = 54		
Percentage Tie Homophily	.295* n = 54	.149 n=50	.192 n = 54	.140 n = 54	
Support	-.262 n = 51	-.072 n = 47	.224* n = 51	.118 n = 51	-.226 n = 51

1. p < .05, ** p < .01

**Table 3:** Ordinal Logistic Regression Results: Perception of Readily-Available Financial Support (n = 51)^†^

**Variable**	**Model 1: Organizational Characteristics**			**Model 2: Inter-Organizational Variables**		
	***Coefficient***	***95% C.I***.	***S.E***.	***Coefficient***	***95% C.I***.	***S.E***.

**Organizational Attributes**						
Non-traditional Health Organization	1.57*	**.34, 2.79**	**.63**	1.55*	**.22, 2.89**	**.68**
Size (Total Employees)	0.00	0.00, 0.00	0.00	0.00	0.00, 0.00	0.00
						
**Partnership Value**						
PV In-Degree				0.03**	**0.01, 0.05**	**0.01**
PV Out-Degree				-0.01	-0.03, 0.01	0.01
Percentage Tie Homophily				-1.13	-3.03, 1.04	1.11

* p < .05, ** p < .01

† Three organizations did not respond to this specific question.
